# Supplementary material for: Modelling Skylarks (Alauda arvensis) to Predict Impacts of Changes in Land Management and Policy: Development and Testing of an Agent-Based Model
Source: PLoS One. 2013 Jun 6;8(6):e65803. doi: 10.1371/journal.pone.0065803 (PMC3675089; doi:10.1371/journal.pone.0065803)
Supplement: Supporting Information S4 — The skylark ODdox as a zipped archive. (ZIP) [file pone.0065803.s004.zip › Skylark_ODdox/globals_vars_0x62.html]

ALMaSS Skylark ODdox: File Members


ALMaSS Skylark ODdox
 2.0


- Main Page
- Related Pages
- Classes
- Files

- File List
- File Members

- All
- Functions
- Variables
- Typedefs
- Enumerations
- Enumerator
- Macros

- a
- b
- c
- d
- e
- f
- g
- h
- i
- j
- k
- l
- m
- n
- o
- p
- r
- s
- t
- v
- w
- x

### - b -

- Breed\_Res\_Thresh1
  : skylarks\_all.cpp
- Breed\_Res\_Thresh2
  : skylarks\_all.cpp
- Breed\_Temp\_Thresh
  : skylarks\_all.cpp


- Generated on Thu Jan 10 2013 13:15:39 for ALMaSS Skylark ODdox by
   1.8.1.1
